# Anaerobic work capacity in cycling: the effect of computational method

**DOI:** 10.1007/s00421-022-05038-7

**Published:** 2022-09-17

**Authors:** Erik P. Andersson, Philipp Bachl, Anna Schmuttermair, Craig A. Staunton, Thomas L. Stöggl

**Affiliations:** 1 Swedish Winter Sports Research Centre, Department of Health Sciences, Mid Sweden University, Östersund, Sweden; 2 School of Sport Sciences, Faculty of Health Sciences, UiT The Arctic University of Norway, Tromsø, Norway; 3 Department of Sport and Exercise Science, University of Salzburg, Salzburg, Austria; 4 Red Bull Athlete Performance Center, Salzburg, Austria

**Keywords:** All-out pacing, Maximal accumulated oxygen deficit method, Metabolic demand, Time trial, Reliability, Supramaximal exercise

## Abstract

**Purpose:**

To compare the anaerobic work capacity (AnWC, i.e., attributable anaerobic mechanical work) assessed using four different approaches/models applied to time-trial (TT) cycle-ergometry exercise.

**Methods:**

Fifteen male cyclists completed a 7 × 4-min submaximal protocol and a 3-min all-out TT (TT_AO_). Linear relationships between power output (PO) and submaximal metabolic rate were constructed to estimate TT-specific gross efficiency (GE) and AnWC, using either a measured resting metabolic rate as a Y-intercept (7 + Y_LIN_) or no measured Y-intercept (7-Y_LIN_). In addition, GE of the last submaximal bout (GE_LAST_) was used to estimate AnWC, and critical power (CP) from TT_AO_ (CP_3´AO_) was used to estimate mechanical work above CP (*W’*, i.e., “AnWC”).

**Results:**

Average PO during TT_AO_ was 5.43 ± 0.30 and CP was 4.48 ± 0.23 W∙kg^−1^. The TT-associated GE values were ~ 22.0% for both 7 + Y_LIN_ and 7-Y_LIN_ and ~ 21.1% for GE_LAST_ (both *P* < 0.001). The AnWC were 269 ± 60, 272 ± 55, 299 ± 61, and 196 ± 52 J∙kg^−1^ for the 7 + Y_LIN_, 7-Y_LIN_, GE_LAST_, and CP_3´AO_ models, respectively (7 + Y_LIN_ and 7-Y_LIN_ versus GE_LAST_, both *P* < 0.001; 7 + Y_LIN_, 7-Y_LIN_, and GE_LAST_ versus CP_3´AO_, all *P* < 0.01). For the three pair-wise comparisons between 7 + Y_LIN_, 7-Y_LIN_, and GE_LAST_, typical errors in AnWC values ranged from 7 to 11 J∙kg^−1^, whereas 7 + Y_LIN_, 7-Y_LIN_, and GE_LAST_ versus CP_3´AO_ revealed typical errors of 55–59 J∙kg^−1^.

**Conclusion:**

These findings demonstrate a substantial disagreement in AnWC between CP_3´AO_ and the other models. The 7 + Y_LIN_ and 7-Y_LIN_ generated 10% lower AnWC values than the GE_LAST_ model, whereas 7 + Y_LIN_ and 7-Y_LIN_ generated similar values of AnWC.

## Introduction

Currently, there is no gold standard method for determining anaerobic capacity (AnC) or anaerobic work capacity (AnWC; i.e., the amount of anaerobic energy that is converted to external work) during whole-body exercise (Noordhof et al. 2010). The most commonly used method for estimating AnC is the linear regression method also referred to as the maximal accumulated oxygen deficit (MAOD) method (Medbø et al. 1988). This method is based on the assumption of a linear relationship between external exercise intensity (e.g., speed or power output) and oxygen uptake (${\dot{\text{v}}\text{o}}_{{2}}$) during submaximal steady-state exercise. Subsequently, the linear relationship can be extrapolated to predict the metabolic requirement in ${\dot{\text{v}}\text{o}}_{{2}}$ equivalents at intensities above maximal ${\dot{\text{v}}\text{o}}_{{2}}$ $\left( {{\dot{\text{v}}\text{o}}_{{{\text{2max}}}} } \right)$, with the difference between the required accumulated VO_2_, and the measured accumulated VO_2_, representing the oxygen deficit. However, due to the different energy equivalents for fat and carbohydrate oxidation, and the effect of submaximal exercise intensity on substrate utilization (Shaw et al. 2014), a linear regression method based on external power output (PO) and metabolic rate (MR) (linear PO-MR regression method) has been suggested to be more appropriate than the traditional MAOD method for determining the required total MR and AnC during supramaximal exercise (Andersson and McGawley 2018; Andersson et al. 2020). Such a method would also enable instantaneous calculation of gross efficiency (GE) during supramaximal exercise through the use of the regression equation to determine the required total MR (Andersson et al. 2020).

Another common method used to determine AnWC and/or AnC is the GE method (Andersson et al. 2020; Noordhof et al. 2011; Serresse et al. 1988). This method requires a single stage of exercise at submaximal steady-state intensity, just below the second ventilatory threshold, as well as a supramaximal exercise bout. For the conventional GE method, the anaerobic contribution to PO is determined as the difference between PO and the aerobic contribution to PO (calculated as aerobic MR multiplied by GE) with AnWC calculated as the anaerobic contribution to PO integrated over time (Noordhof et al. 2011). Thus, AnWC is dependent on both the AnC and GE. Although a not common practice, the GE method can easily determine AnC. This is performed by dividing supramaximal PO by GE to calculate the required total MR, with anaerobic MR being calculated as the difference between required total MR and aerobic MR, which when integrated over time represents the AnC (Andersson et al. 2020).

The obvious advantage of the GE versus the linear PO-MR regression method is that it is far less time-consuming, as it only requires one submaximal stage compared to the linear regression method which requires ~ 5–10 submaximal stages (Noordhof et al. 2011). However, one assumption with the GE method that differs from the linear PO-MR regression method is that GE is PO independent, which only is the case for the linear PO-MR regression method if the Y-intercept value in the regression is zero (Andersson et al. 2020). In addition, the GE method can be converted to a linear PO-MR equation where the slope represents the reciprocal value of GE combined with a zero Y-intercept (Andersson et al. 2020). Although the traditional MAOD method is inappropriate for determining AnWC (i.e., anaerobically attributable mechanical work) (Medbø et al. 1988), the supramaximal instantaneous GE calculated from the linear PO-MR regression method can be used to determine the anaerobically attributable PO and, thus, AnWC during supramaximal exercise (Andersson et al. 2020). One clear difference between the GE method and the linear PO-MR regression method is that the GE method is solely based on a constant GE. In contrast, the linear PO-MR regression method assumes an increasing GE if the Y-intercept of the linear PO-MR regression is positive, which is the case for cycle-ergometry exercise (Ettema and Lorås 2009). Therefore, the associated GE during supramaximal cycle exercise would likely be higher for the linear PO-MR regression method compared to the GE method and result in higher values of estimated AnWC/AnC for the latter method.

Another concept that can be used to differentiate between aerobic and anaerobic power contributions to external PO is the critical power (CP) method. With the CP concept, the CP threshold equals the maximal PO that is generated by primarily aerobic energy sources and can, at least in theory, be maintained indefinitely, whereas the exercise duration above CP is finite (Vanhatalo et al. 2007, 2011). For determining CP, the hyperbolic relationship between exercise duration and PO for maximal exercise first needs to be established. This requires participants to complete ≥ 4 separate maximal tests to exhaustion at several different fixed power outputs (Vanhatalo et al. 2011). In contrast to this traditional and time-consuming approach of determining CP, a modified version of the CP protocol has been proposed, which involves just a single 3-min all-out time trial (TT) (Vanhatalo et al. 2007). Due to depletion of the anaerobic energy reserve during the initial stages of exercise (≤ 2 min of exercise), the average PO during the final 30 s (referred to as “end power”) of the 3-min all-out test is considered to represent CP (Vanhatalo et al. 2007). With the CP method, the total mechanical work above critical power (*W′*) is calculated as PO above CP integrated over time and is often referred to as a marker of AnWC (Hill 1993; Morton 2006). However, referring to *W′* as a pure AnWC may be incorrect as it also can include aerobically attributable work (Vinetti et al., 2017, 2019). The aerobic component of *W′* is, though, compensated by the assumption that PO at CP is exclusively supplied by aerobic metabolism at the onset of exercise (i.e., neglecting the primary component of ${\dot{\text{v}}\text{o}}_{{2}}$ kinetics) with the implication that *W’* can represent a valid AnWC.

In a previous study by Hill and Smith (1993), the *W′* estimate of AnWC was compared with an adopted MAOD method, with similar average estimates of AnWC found between the two methods. However, these estimates of AnWC were not perfectly correlated (*r* = 0.55 and *r* = 0.78 for females and males, respectively). Although, Hill and Smith (1993) found comparable average values of AnWC for the CP and GE methods, a more recent study by Dekerle et al. (2006) showed a poor agreement between *W′* based on CP and AnWC during a 90-s all-out cycling test, with AnWC being calculated as the difference between external PO and aerobically attributable PO integrated over time. Several possible factors could explain the disagreement in AnWC determined with the CP (i.e., *W′*) and “MAOD”/”GE” methods used in previous studies (Dekerle et al. 2006; Hill and Smith 1993). One main factor for these divergences is likely related to the fact that aerobically attributable PO is approximated with the CP model without any physiological assessment. However, the *W’* measure of AnWC based on the CP concept can attain a lower, similar, or even higher, value than the AnWC based on the GE method because there can be aerobically attributable PO above CP up to the maximal aerobically attributable PO (i.e., PO at ${\dot{\text{v}}\text{o}}_{2\max }$) (Vinetti et al. 2017, 2019). For instance, if the average fractional utilization of ${\dot{\text{v}}\text{o}}_{2\max }$ (as a percentage of ${\dot{\text{v}}\text{o}}_{{{\text{2max}}}}$) during a maximal effort is the same as the corresponding ${\dot{\text{v}}\text{o}}_{{2}}$ requirement at CP (as a percentage of ${\dot{\text{v}}\text{o}}_{{{\text{2max}}}}$), *W’* would, in theory, attain a similar value as AnWC based on the GE method.

Only a few studies have compared the GE and MAOD computational methods for determining AnC during cycle ergometry (Noordhof et al. 2011) and treadmill roller-skiing exercise (Andersson and McGawley 2018; Andersson et al. 2020). In each of these studies, the disagreement between AnC estimates across different models was found to be considerable and suggests that different models should not be used interchangeably (Andersson et al. 2020; Noordhof et al. 2011). However, there is sparse research that has compared the *W′* estimate of “AnWC” (based on the CP method) against the AnWC estimated with the MAOD and/or GE methods (Dekerle et al. 2006; Hill and Smith 1993). To our knowledge, there is only one study that has analyzed the agreement between *W′* based on CP against the estimated accumulated oxygen deficit based on a linear ${\dot{\text{v}}\text{o}}_{{2}}$-PO regression method during a 3-min all-out time trial (Muniz-Pumares et al. 2016). One limitation of this previous study was that the agreement between the two methods was assessed by correlational analysis (Bland and Altman 1999) and that different units were correlated, i.e., correlating AnC (in oxygen equivalents) with AnWC (Winter and Fowler 2009).

Given the limited number of methodological studies, the primary aim of the current study was to compare estimates of AnWC and/or AnC generated during a 3-min all-out time trial (TT_AO_) using four different models: the 7 × 4-min linear regression method based on PO and MR with, and without, the inclusion of a resting baseline MR value as a Y-intercept (7 + Y_LIN_ and 7-Y_LIN,_ respectively); the GE method using the last exercise intensity (GE_LAST_); and the CP method based on the 30-s average end power as CP (CP_3´AO_).

The hypotheses of the current study were as follows:

- 1) the GE_LAST_ model would generate the highest values of AnWC/AnC compared to the other models tested; and
- 2) there would be disagreement in *W’*/AnWC between the CP_3´AO_ versus the 7 + Y_LIN_, 7-Y_LIN_, and GE_LAST_ models.

## Methods

### Participants

Fifteen highly trained male cyclists (mean ± standard deviation: age: 28.0 ± 4.7 yr., body mass: 78.5 ± 7.7 kg, stature: 183.5 ± 7.0 cm) were recruited for this study (Tier 3–4, McKay et al. 2022). During the 24 h prior to testing, participants were instructed to perform ≤ 2 h of low-intensity exercise and refrain from intake of alcohol. The last regular meal had to be eaten at least 3 h pre-test; however, a small snack (e.g., an energy bar) could be consumed 1–2 h before the test. The participants could consume water ad libitum, but no intake of any food was allowed during the test. The study was approved by the ethical review board of the University of Salzburg (EK-GZ: 05/2020). All participants were fully informed about the nature of the study and provided written consent before the first test. Exclusion criteria were any of the following: ${\dot{\text{v}}\text{o}}_{{{\text{2peak}}}}$ < 55 ml kg^−1^ min^−1^; no previous experience of laboratory cycle-ergometry tests; and injury or illness.

### Equipment, measurements, and testing procedures

**Fig. 1:**
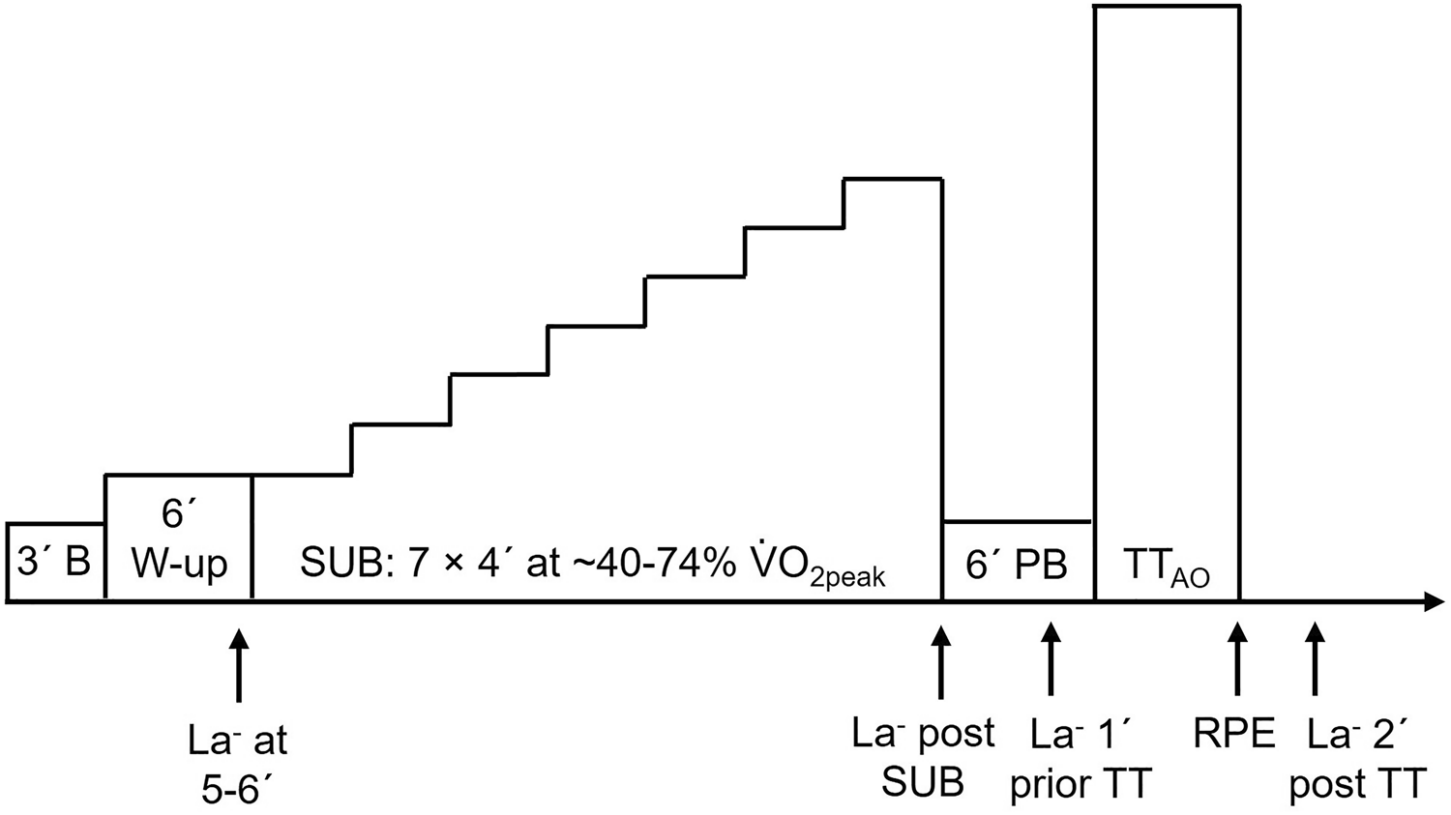
A schematic overview of the testing protocol where participants performed a 3-min all-out time trial (TT_AO_). After a 3-min baseline measure of oxygen consumption (3´ B) and a 6-min warm-up (6´ W-up), the 7 × 4-min submaximal exercise stages were performed and followed by a 6-min passive break (6´ PB). Capillary blood samples for the determination of blood lactate concentration (La^−^) were collected prior to and immediately after the submaximal stages and 1-min before and 2-min after the TT. Abbreviations: ´, minute; ${\dot{\text{v}}\text{o}}_{{{\text{2peak}}}}$, peak oxygen consumption; SUB, sub-maximal

The participants used their own clothes, cycling shoes, cleats, and pedals. All testing was performed on a bike designed for TT tests (Monark LC7TT, Monark Exercise AB, Vansbro, Sweden) equipped with road race handlebars and standard shifting mechanics (Shimano Ultegra 11 Speed, Shimano Inc., Osaka, Japan). Cycling PO was logged continuously as second-by-second data. The bike was fully adjustable to the rider’s preferences (i.e., seat tube length, saddle-setback, the height of the stem, and forward length of the stem). The participant’s stature and body mass were measured before the first test using an electronic scale (Seca 764, Hamburg, Germany). Respiratory variables were measured using a Cosmed Quark CPET mixing chamber ergospirometry system (Cosmed Srl, Rome, Italy) with raw data as 10-s values. This set-up was used to provide valid and reliable metabolic measurements, especially at high ventilation rates in highly trained athletes (Nieman et al. 2013; Winkert et al. 2020). Prior to each test, the oxygen (O_2_) and carbon dioxide (CO_2_) sensors were calibrated using a two-point calibration procedure with ambient air conditions and the anticipated expiratory gas percentages using a known calibration gas containing 15% O_2_ and 5% CO_2_ (UN 1950 Aerosols, Cortex Biophysik GmbH, Leipzig, Germany). The flow volume was calibrated using a 3-L syringe (M9424, Medikro Oy, Kuopio, Finland). Blood lactate concentration was measured via ear-lobe capillary samples (20 μL) that were subsequently analyzed using a Biosen S-line (EKF diagnostic GmbH, Magdeburg, Germany). The Biosen S-line was calibrated with a known standard solution of 12 mmol L^−1^. The blood samples were collected at the final minute of the 6-min warm-up, directly after completion of the submaximal protocol, 1 min prior to the start of the TT, and 2 min after the TT (see Fig. 1). Heart rate was measured continuously throughout the test using a heart rate belt (Wahoo Kickr, Wahoo Fitness, Atlanta, GA, United States) that was connected to the ergospirometry system.

A 3-min baseline respiratory sample was measured prior to the warm-up with the participant being seated upright on the bicycle with both feet resting on the pedals. The baseline respiratory sample was preceded by a 3-min seated rest. The warm-up protocol consisted of 6-min at ~ 40% of peak ${\dot{\text{v}}\text{o}}_{{2}}$ $\left( {{\dot{\text{v}}\text{o}}_{{{\text{2peak}}}} } \right)$ and started immediately following the baseline respiratory sample. The warm-up was directly followed by the submaximal protocol that consisted of seven 4-min stages (7 × 4) that were performed between ~ 40–74% of ${\dot{\text{v}}\text{o}}_{{{\text{2peak}}}}$ with ~ 6% increment per stage. This was followed by a 6-min passive rest and the 3-min TT_AO_. The TT_AO_ concept was utilized to establish CP, using the 3-min all-out method, which allows for an estimation of AnWC (Burnley et al. 2006; Vanhatalo et al. 2007). During the TT_AO_, heart rate, PO, and elapsed time were not visible to the participant and the average PO of the last 30 s of the all-out effort was used to calculate CP according to previous studies (Burnley et al. 2006; Vanhatalo et al. 2007). Furthermore, participants were instructed to provide maximal effort (“no holding back”) from the beginning of the TT_AO_. The PO for the warm-up and submaximal stages was fixed and cadence independent whereas, for the TT_AO_, the PO was regulated freely by the athlete via the bike’s shifters and was cadence dependent. The cadence was self-selected during the submaximal stages and TT_AO_. The cadence for the submaximal exercise was determined as the average of the final minute of each stage. Peak cadence and peak PO during TT_AO_ were determined as the highest respective 5-s moving average. A schematic overview of the testing procedure is presented in Fig. 1. During the final minute of the last submaximal stage and immediately after the TT, the participant reported a rating of perceived exertion using the Borg 6–20 scale (Borg 1982). All participants were familiar with submaximal and maximal cycle ergometry efforts. The submaximal power outputs were selected based on previous test results.

### Processing of respiratory data

Respiratory and heart rate data were collected continuously during the submaximal exercise and the TT_AO_. To enable a higher resolution of respiratory data during TT_AO_ (i.e., to obtain a more dynamic respiratory response), raw 10-s respiratory data were transferred to second-by-second data (using piecewise constant interpolation for each 10-s value) and smoothed using a 9-s counterbalanced moving average (i.e., ± 4-s time window for smoothing), which was conducted twice. For the start-point of the TT_AO_, a gradual increase in the smoothing function time window was used up to the fifth second, whereafter the 9-s counterbalanced moving average was used. For the endpoint of TT_AO_, the same principle was employed, but with a gradual decrease in the smoothing time window over the last four seconds. The highest 20-s moving average during the TT_AO_ was used to calculate ${\dot{\text{v}}\text{o}}_{{{\text{2peak}}}}$, with peak RER taken over the same period. In addition, ${\dot{\text{v}}\text{o}}_{{{\text{2peak}}}}$ was converted to a peak aerobic metabolic rate [Eq. 1] using an RER of 1.00 (i.e., assuming 100% carbohydrate utilization at ${\dot{\text{v}}\text{o}}_{{{\text{2peak}}}}$). Peak heart rate was considered the highest 10-s average value.

### Calculations

#### Submaximal exercise

Energy expenditure was calculated from ${\dot{\text{v}}\text{o}}_{{2}}$ and RER (${\dot{\text{v}}\text{co}}_{{2}} \,{\dot{\text{v}}\text{o}}_{{2}}^{ - 1}$) according to the equation published by Weir (1949) and then converted into a MR. The MR was based on the average ${\dot{\text{v}}\text{o}}_{{2}}$ in L min^−1^ and RER values (≤ 1.00) during the final minute of each stage of the submaximal exercise protocol.

1 $$MR [W]=\frac{4184{\dot{(V}O}_{2}\left(1.1RER+3.9\right))}{60}$$

GE was calculated as:

2 $$GE=\frac{PO [W]}{MR [W]}$$

Net efficiency was calculated as:

3 $$Net efficiency=\frac{PO [W]}{MR-{MR}_{BL} [W]}$$

where MR_BL_ is the baseline MR calculated from an average 3-min baseline ${\dot{\text{v}}\text{o}}_{{2}}$ and RER measurement with the participant seated on the cycle ergometer with no pedaling. Delta efficiency was calculated as the reciprocal value of the slope of the PO-MR regression equation. Neither net efficiency nor delta efficiency was used for estimating AnWC/AnC.

#### Estimating AnC, AnWC, and supramaximal GE

A linear relationship between PO (W) and MR (W) during the final minute of each of the 7 × 4-min submaximal stages was derived for each participant with the baseline MR (i.e., the MR at zero speed) included (7 + Y_LIN_) or excluded (7-Y_LIN_) from the model. In the latter case, the Y-intercept was based on all data points in the regression but excluding the baseline resting value of MR. The 7 + Y_LIN_ and 7-Y_LIN_ regression equations were used to estimate the required instantaneous MR during the 3-min TT (MR_TT_req_) at each 1-s time point. Submaximal GE from the last submaximal stage (GE_LAST_) was also used to estimate the MR_TT_req_ at each 1-s time point of the TT. MR_TT_req_ was calculated by dividing instantaneous PO with GE_LAST_. For illustrative purposes, GE_LAST_ was converted to a PO-MR linear equation, where the slope was calculated as the reciprocal value of GE_LAST_, with a Y-intercept of zero due to the constant GE assumption for the GE_LAST_ model. The instantaneous second-by-second GE values during the TT were also calculated for the 7 + Y_LIN_ and 7-Y_LIN_ models (GE_REG_) as instantaneous PO divided by the instantaneous required MR calculated from the regression equation.

For the 7 + Y_LIN_, 7-Y_LIN_, and GE_LAST_ models, the instantaneous anaerobic MR (MR_AN_) at each 1-s time-point (*t*) of the TT could then be expressed as:

4 $${MR}_{AN,t}[W]={MR}_{TT\_req,t}- {MR}_{AE,t}$$

where MR_AE_ is the aerobic MR calculated according to Eq. 1 and using an RER of 1.00 (i.e., assuming 100% carbohydrate utilization during the TT).

For all three models, the total anaerobic energy production (i.e., AnC [kJ kg^−1^]) was calculated by integrating MR_an_ over the 3-min TT.

Anaerobic PO contribution (PO_AN_cont_) (i.e., PO attributable to MR_AN_) at each 1-s time point (*t*) of the TT was calculated for the 7 + Y_LIN_ and 7-Y_LIN_ models as:

5 $${PO}_{A{N}_{cont},t}={{PO}_{TT,t}\left[W\right]-(MR}_{AE,t}\times {GE}_{REG,t})$$

where PO_TT_ is the PO during the TT.

For GE_LAST_, the same equation was used but with the exception that GE_REG_ was changed to GE_LAST_.

The AnWC in joules (J) was calculated for the 7 + Y_LIN_, 7-Y_LIN_, and GE_LAST_ models by integrating the model-specific PO_AN_cont_ (W) over the TT duration (s).

For the CP_3´AO_ model, the total mechanical work above CP (i.e., *W’*) was calculated as the PO above CP integrated over time. The CP was determined as the average PO of the last 30 s of TT_AO_ (Vanhatalo et al. 2007). In the current study, *W’* based on CP_3´AO_ has been referred to as an “AnWC”, because *W’* is usually referred to as a surrogate marker of AnWC (Hill, 1993; Morton, 2006).

To calculate the average supramaximal GE during the TT for both the 7 + Y_LIN_ and 7-Y_LIN_ models, the estimated instantaneous GE of the 3-min TT was calculated as instantaneous PO divided by instantaneous MR_TT_req_ (derived from the linear PO-MR regression equation) and expressed as an average TT value.

### Statistical analyses

All statistical tests were processed using Office Excel 2016 (Microsoft Corporation, Redmond, WA, USA) and the Statistical package for the social sciences (SPSS 25, IBM Corp., Armonk, NY, USA). The level of statistical significance was set at *α* = 0.05. Normality of data was confirmed by visual inspection of Q–Q plots and histograms together with the Shapiro–Wilk analysis. Accordingly, data are presented as mean ± standard deviation, except in the case of peak heart rate and rating of perceived exertion where data are presented as median and interquartile range. In addition, the different AnWC estimates are presented as mean and 95% confidence interval. The PO-MR relationships for the 7 + Y_LIN_ and 7-Y_LIN_ models were assessed using linear regression analyses. One-way repeated measures ANOVA tests were used to compare GE and net efficiency between the seven submaximal stages as well as the GE, required MR, and AnC associated with the TT. A paired *t* test was used to compare the linear regression coefficients for the 7 + Y_LIN_ and 7-Y_LIN_ models. The precision of the two linear regression equations was assessed with the standard error of the estimate. For the ANOVA tests, the assumption of sphericity was assessed using Mauchly’s test. For violated sphericity, a Greenhouse–Geisser correction of the degrees of freedom was used (epsilon ≤ 0.75). Eta-squared effect size was reported for the one-way repeated measures ANOVA tests. Bonferroni *α* corrections were applied to all ANOVA tests.

The mean difference ± 95% limits of agreement for the comparison of the AnWC estimates were evaluated using Bland–Altman calculations (Bland and Altman 1999). The mean difference was tested with a paired-sample *t*-test and the standardized mean difference (Hedges’ *g*_*av*_, effect size [ES_*Hg_av*_]) was reported according to the equations presented by Lakens (2013). In addition, the methodological error was evaluated via the overall standard error of measurement calculated as the square root of the within-groups mean square error term in the repeated measures ANOVA and the absolute typical error for the separate pair-wise comparisons. The typical error was also expressed as a percentage, i.e., as a percentage of the grand mean.

## Results

### Physiological and cadence responses to submaximal exercise

The PO, cadence, physiological responses, and two types of efficiency (i.e., GE and net efficiency) at the seven submaximal stages are shown in Table 1. The blood lactate concentrations at the fifth minute of the warm-up, and immediately after the submaximal exercise, were 1.0 ± 0.2 and 2.7 ± 0.5 mmol L^−1^, respectively. The rating of perceived exertion value at the last minute of the final submaximal stage was 15 (interquartile range = 15–16). The GE increased from the first to the last submaximal stage prior to the TT (F_2,32_ = 42.8, *P* < 0.001, eta-squared effect size = 0.754), whereas the net efficiency remained unchanged during all the submaximal stages prior to the TT (F_3,36_ = 2.5, *P* = 0.085, eta- squared effect size = 0.151).

**Table 1:** Mean ± standard deviation of power outputs, cadences, heart rates, cardiorespiratory variables, and efficiencies associated with the seven submaximal stages (SUB_1-7_) of cycle ergometry exercise, as well as the seated resting baseline (BL_REST_) data

	BL_REST_	SUB_1_	SUB_2_	SUB_3_	SUB_4_	SUB_5_	SUB_6_	SUB_7_
Power output (W kg^−1^)	0	1.62 ± 0.11	1.93 ± 0.14	2.24 ± 0.16	2.54 ± 0.18	2.85 ± 0.20	3.16 ± 0.23	3.47 ± 0.25
Cadence (rev min^−1^)	–	68 ± 6	70 ± 6	73 ± 6	76 ± 6	79 ± 6	82 ± 5	84 ± 6
Heart rate (% of TT_peak_)	40 ± 5	61 ± 3	65 ± 3	70 ± 3	74 ± 3	79 ± 3	84 ± 2	89 ± 2
MR_AE_ (W∙kg^−1^)	2.0 ± 0.4	8.8 ± 0.8	9.8 ± 0.9	11.1 ± 1.0	12.4 ± 1.0	13.7 ± 1.1	15.0 ± 1.1	16.5 ± 1.4
MR_AE_ (% of MR_AE_peak_)	9 ± 2	40 ± 4	44 ± 4	50 ± 5	56 ± 4	62 ± 5	68 ± 5	74 ± 5
$\dot{\mathrm{V}}$_E_ (L min^−1^)	16.7 ± 4.6	52.2 ± 6.9	56.0 ± 7.4	63.0 ± 8.4	71.7 ± 9.9	79.4 ± 10.7	89.2 ± 11.6	100.7 ± 14.8
$\dot{\mathrm{V}}$_E_ $\dot{\mathrm{V}}\mathrm{O}$_2_^–1^	35.1 ± 4.7	25.5 ± 2.0	24.4 ± 1.8	24.4 ± 1.7	24.8 ± 2.1	25.0 ± 1.9	25.6 ± 1.9	26.4 ± 2.4
RER ($\dot{\mathrm{V}}$ CO_2_∙$\dot{\mathrm{V}}$ O_2_^−1^)	0.85 ± 0.08	0.87 ± 0.04	0.86 ± 0.04	0.87 ± 0.04	0.88 ± 0.04	0.89 ± 0.04	0.90 ± 0.04	0.91 ± 0.04
Gross efficiency (%)	–	18.5 ± 1.5	19.7 ± 1.4	20.3 ± 1.4	20.5 ± 1.2	20.9 ± 1.2	21.0 ± 1.2	21.1 ± 1.1
Net efficiency (%)	–	24.0 ± 2.1	24.8 ± 2.1	24.7 ± 1.9	24.5 ± 1.5	24.6 ± 1.9	24.3 ± 1.7	24.0 ± 1.6

Abbreviations: *TT*_*peak*_ time trial peak value, *MR*_*AE*_ aerobic metabolic rate, *MR*_*AE_peak*_ peak aerobic metabolic rate during the time trial, $\dot{V}$_*E*_ ventilation rate, $\dot{V}$_*E*_*∙*$\dot{V}O$_*2*_^*–1*^ ventilatory equivalent for oxygen; RER, respiratory exchange ratio

### Performance, cadence, and physiological responses during TT_AO_

The average PO during TT_AO_ was 5.43 ± 0.30 W kg^−1^ at an average cadence of 92 ± 9 rev min^−1^. The peak PO was 9.3 ± 1.2 W kg^−1^ and the peak cadence was 127 ± 19 rev∙min^−1^. The average cadence during the last 30 s of the TT_AO_ was 90 ± 14 rev min^−1^. The ${\dot{\text{v}}\text{o}}_{{{\text{2peak}}}}$ was 64 ± 6 ml kg^−1^ min^−1^ (5.0 ± 0.6 L min^−1^) at an RER of 1.10 ± 0.10. The average ${\dot{\text{v}}\text{o}}_{{2}}$ was 51 ± 6 ml kg^−1^ min^−1^, which resulted in an average fractional utilization of 80 ± 3% of ${\dot{\text{v}}\text{o}}_{{{\text{2peak}}}}$. The peak heart rate was 183 (interquartile range = 179–186) beats min^−1^. The blood lactate concentrations measured 1 min prior to the TT and 2 min after the TT were 2.1 ± 0.5 and 11.5 ± 2.6 mmol L^−1^. The median rating of perceived exertion value immediately after the TT was 20 (interquartile range = 19–20).

### Comparison between the different models used to estimate AnWC/AnC

**Fig. 2:**
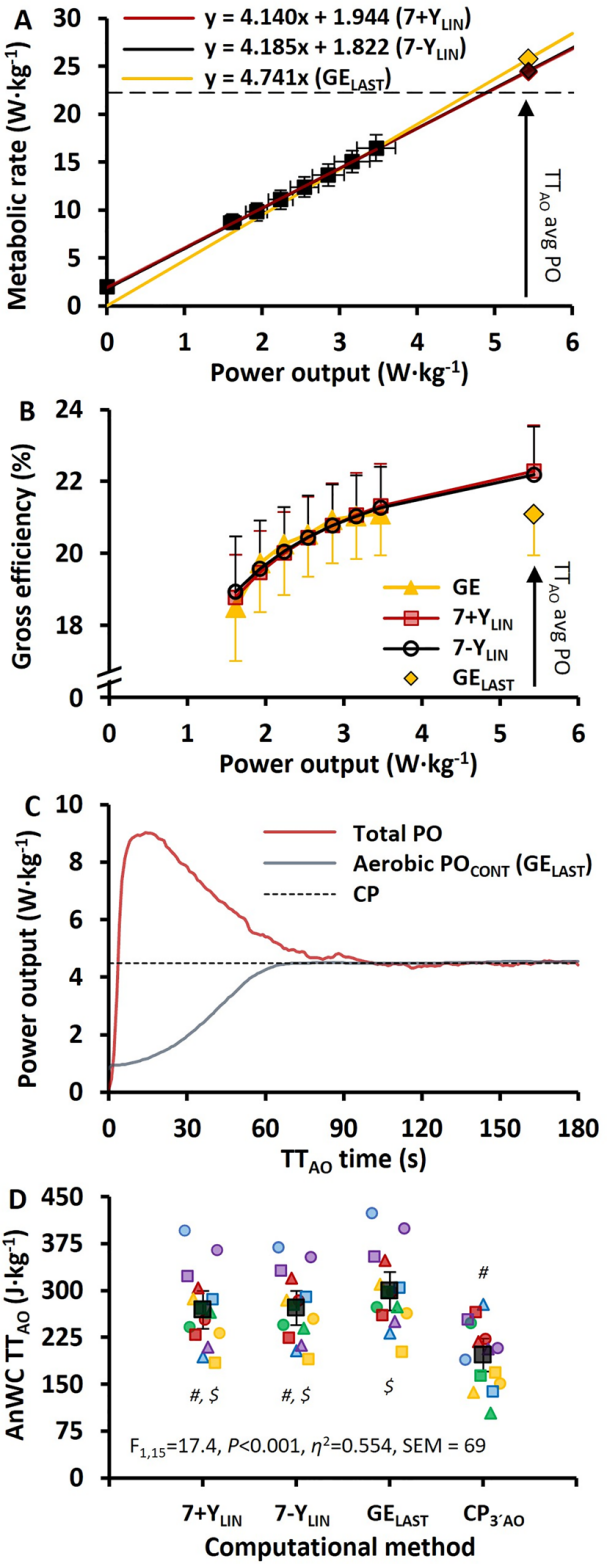
Data based on 7 stages of submaximal exercise followed by a 3-min supramaximal all-out time trial (TT_AO_) (A-D). **A** The two regression models between mean ± standard deviation power output (PO) and metabolic rate during 7 × 4-min stages of continuous cycle ergometry exercise and the regression line calculated based on the gross efficiency (GE) from the last submaximal stage (GE_LAST_) together with the estimated total metabolic requirements (diamonds) at the average PO attained during the 3-min time trial. The red line is the linear relationship when using a Y-intercept (7 + Y_LIN_) for baseline metabolic rate, the black solid line when excluding a Y-intercept value (7-Y_LIN_), and the yellow line is the regression line based on GE_LAST_ (i.e., with the slope being the reciprocal value of GE); **B** GE as mean ± standard deviation for the seven 4-min stages of submaximal cycling and GE calculated from the two regression equations (i.e., 7 + Y_LIN_ and 7-Y_LIN_) for the submaximal stages and the TT; (C) Total PO (PO) and aerobic power contribution (based on GE_LAST_) presented as second-by-second average time-trial data and the estimated critical power (CP); (D) The mean anaerobic work capacity (AnWC) and 95% confidence interval together with individual data (colored symbols). *F *values, *P* values, eta-squared effect size (*n*^2^), and standard error of measurement (SEM) were obtained with a repeated measures ANOVA. ^#^Significantly different from GE_LAST_, *P* ≤ 0.001; ^*$*^Significantly different from CP, *P* ≤ 0.030

The mean PO-MR regression lines that were based on the seven submaximal stages for the 7 + Y_LIN_ and 7-Y_LIN_ models and calculated from GE based on the last submaximal stage (GE_LAST_) with extrapolation up to the TT are displayed in Fig. 2A. The mean ± standard deviation values of directly measured GE and the GE calculated from the 7 + Y_LIN_ and 7-Y_LIN_ models are displayed in Fig. 2B. The mean instantaneous total PO, aerobic contribution to PO (based on GE_LAST_), and CP are presented in Fig. 2C, with the total PO above CP integrated over time representing the *W’* (i.e., “AnWC”) estimated with the CP_3´AO_ model. The CP was 4.5 ± 0.2 W kg^−1^, which was equivalent to 93 ± 10%, 93 ± 9%, and 96 ± 10% of the peak aerobic MR (i.e., the ${\dot{\text{v}}\text{o}}_{{{\text{2peak}}}}$ expressed as a MR) for the 7 + Y_LIN_, 7-Y_LIN_, and GE_LAST_ models. The mean AnWC and 95% confidence interval together with individual data (colored symbols) are presented in Fig. 2D. It can be noted that the AnWC was considerably lower for the CP_3´AO_ model compared to 7 + Y_LIN_, 7-Y_LIN_, and GE_LAST_ models, while the GE_LAST_ generated the highest values of AnWC.

The data presented in Table 2 show that the standard error of the estimate was larger for the 7 + Y_LIN_ versus the 7-Y_LIN_ regression model. The regression slope and Y-intercept values were similar for the 7 + Y_LIN_ and 7-Y_LIN_ models, which resulted in similar average GE values during the TT. The average GE during the TT was ~ 0.9 percentage points lower for the GE_LAST_ than the 7 + Y_LIN_ and 7-Y_LIN_ models.

**Table 2:** Mean ± standard deviation of slope, delta efficiency, Y-intercept, coefficient of determination (*r*^2^), standard error of estimate (SEE) for the two linear regression models, and gross efficiency, metabolic requirement, and anaerobic capacity during the 3-min all-out cycle time trial for the three different models of estimating the anaerobic capacity

Method of calculation							
	7 + Y_LIN_	7-Y_LIN_	GE_LAST_	Test statistic	*P*-value	ES	SEM
Slope (MR per PO [W kg^−1^])	4.14 ± 0.28	4.19 ± 0.38	–	–	*P* = 0.478	*Hg*_*av*_ = − 0.1	−
Delta efficiency (%)	24.2 ± 1.6	24.1 ± 2.3	–	–	*P* = 0.641	*Hg*_*av*_ = 0.1	−
Y-intercept (W kg^−1^)	1.94 ± 0.50	1.82 ± 1.00	–	–	*P* = 0.449	*Hg*_*av*_ = 0.1	−
r^2^	0.996 ± 0.003	0.992 ± 0.007	–	–	*P* = 0.003	*Hg*_*av*_ = 0.7	−
SEE (W kg^−1^)	0.28 ± 0.10	0.25 ± 0.08	–	–	*P* = 0.070	*Hg*_*av*_ = 0.3	−
GE_TT_avg_ (%)	22.0 ± 1.2^*^	22.0 ± 1.3^*^	21.1 ± 1.1	F_2,28_ = 35.9	*P* < 0.001	η^2^ = 0.720	0.3
MR_TT_req_ (W kg^−1^)	24.4 ± 1.8^*^	24.5 ± 1.8^*^	25.8 ± 2.0	F_2,28_ = 50.5	*P* < 0.001	η^2^ = 0.783	0.4
MR_TT_req_ (% of MR_ae_peak_)	110 ± 6^*^	111 ± 6^*^	116 ± 7	F_2,28_ = 48.2	*P* < 0.001	η^2^ = 0.775	2
AnC (kJ kg^−1^)	1.17 ± 0.28^*^	1.19 ± 0.24^*^	1.42 ± 0.30	F_2,28_ = 50.5	*P* < 0.001	η^2^ = 0.783	0.08

Abbreviations: 7 + Y_LIN_ and 7-Y_LIN_, the 7 × 4-min linear models with the baseline metabolic rate as a Y-intercept either included (7 + Y) or excluded (7-Y), GE_LAST_, the gross efficiency model based on the last submaximal stage, *MR* metabolic rate, *PO* power output, *GE*_*TTavg*_ average GE during the TT, *MR*_*TTreq*_ required metabolic rate during the TT, *MR*_*ae_peak*_ peak aerobic metabolic rate during the TT, *AnC* anaerobic capacity, *Hg*_*av*_ Hedge’s *g*_*av*_ effect size. *F* values, *P* values, and eta-squared effect size (η^2^) were obtained by a one-way ANOVA. ^*^Statistically significantly different from GE_LAST_ (*P* < 0.001)

**Fig. 3:**
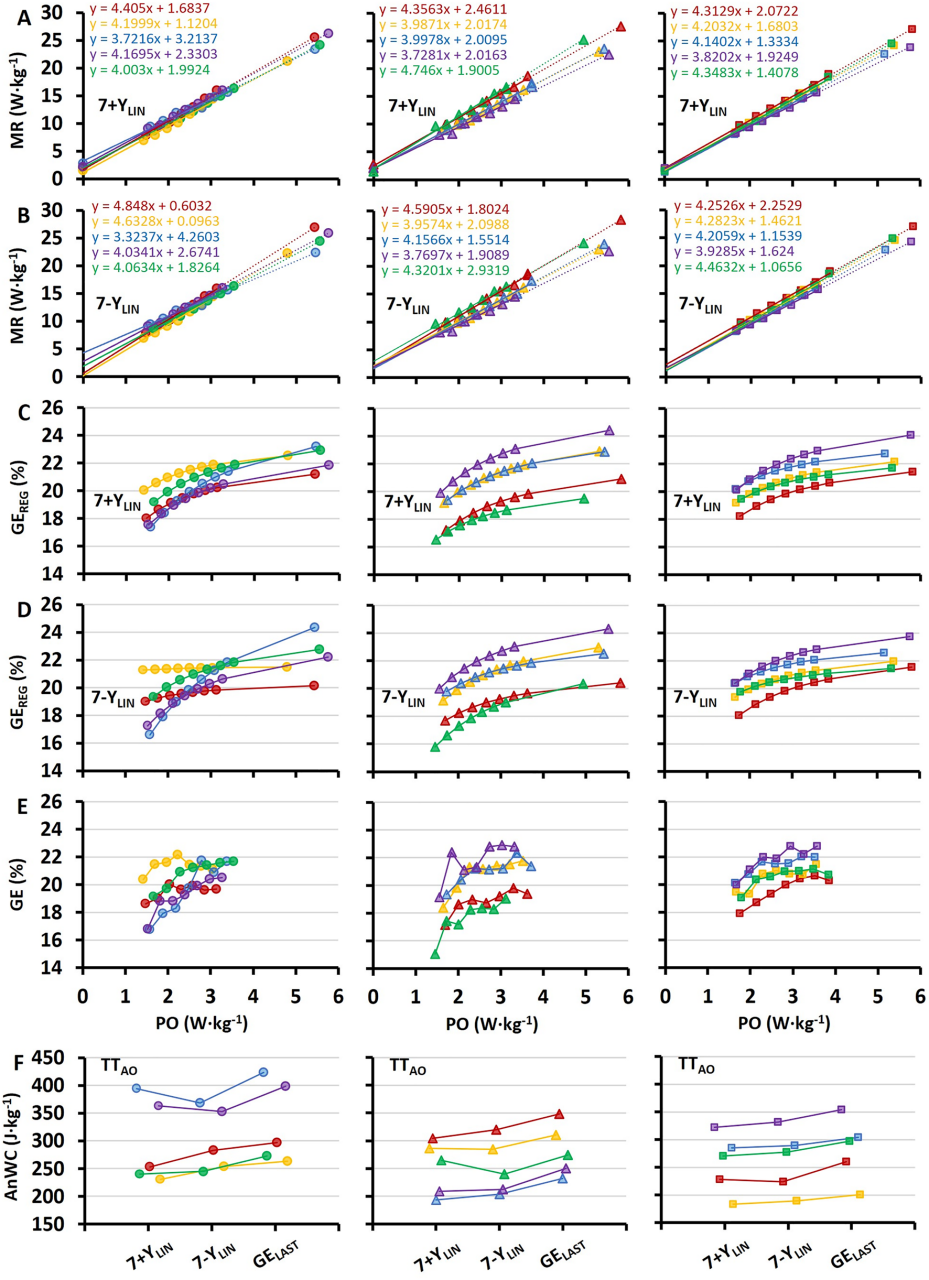
Individual data (*N* = 15, 5 in each of the three horizontal panels) based on 7 × 4-min of submaximal exercise followed by a 3-min supramaximal all-out TT (TT_AO_). Regressions for metabolic rate plotted against cycling power output (PO) based on the 7 × 4-min submaximal stages and the extrapolation up to the average PO during TT_AO_ including a Y-intercept value, i.e., baseline metabolic rate, (7 + Y_LIN_) (**A**) and when excluding a Y-intercept value (7-Y_LIN_) in the respective regressions (**B**). Gross efficiency calculated from the two linear regression equations (GE_REG_) for the submaximal stages and the TT, with values from 7 + Y_LIN_ in C and values from 7-Y_LIN_ in **D**. Directly measured values of GE based on the seven submaximal stages (**E**). Individual values of anaerobic work capacity (AnWC) calculated with the three different methods (F), where the 7 + Y_LIN_ and 7-Y_LIN_ are the two linear models, and the GE_LAST_ model is based on the GE value from the last submaximal stage

**Fig. 4:**
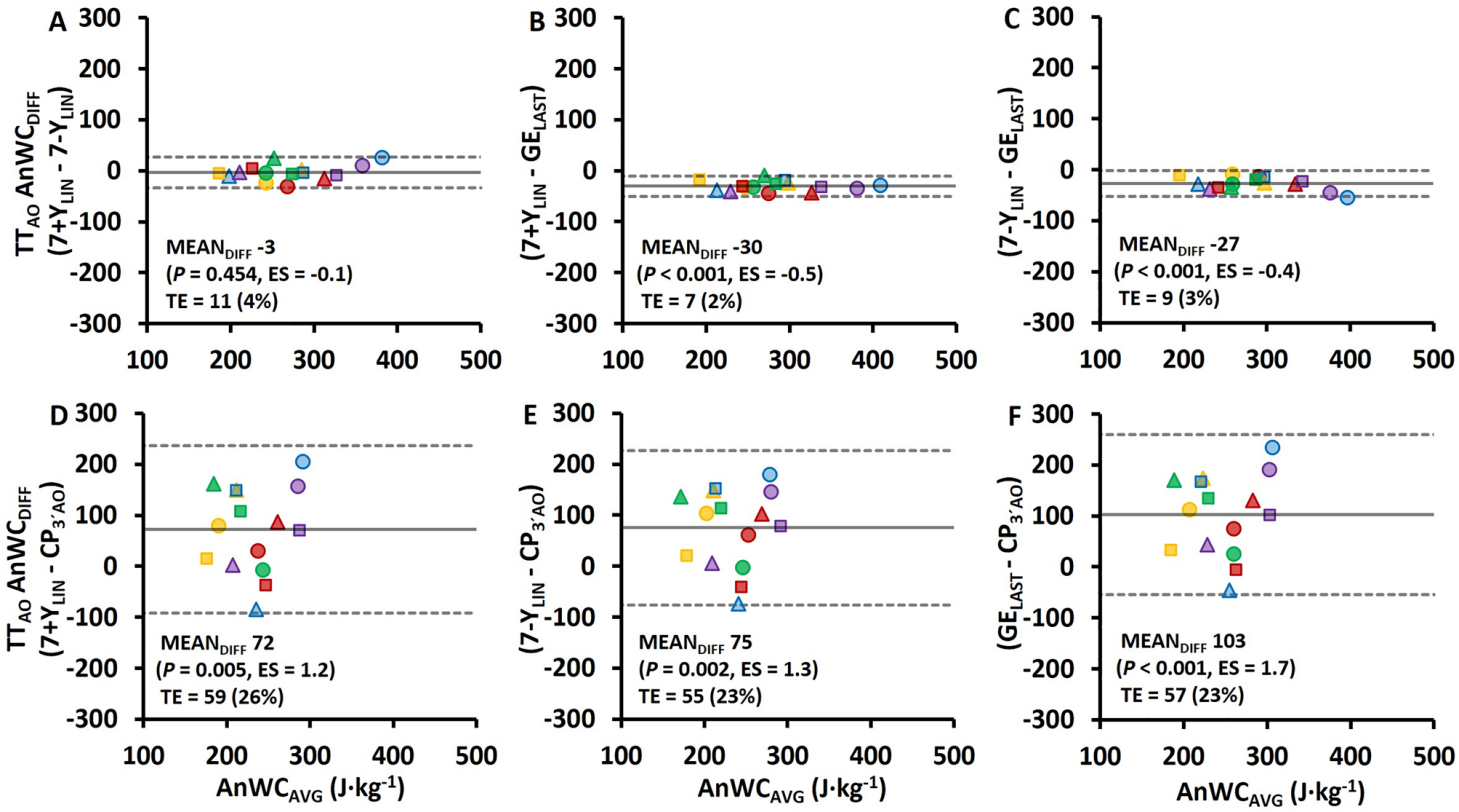
Bland–Altman plots for the four various models of estimating anaerobic work capacity (AnWC) associated with the 3-min all-out cycle time trial (TT_AO_) in **A**–**F**. Bland–Altman plots represent the mean difference (MEAN_DIFF_) in the AnWC ± 95% (1.96 standard deviations) limits of agreement between the methods. Abbreviations: AnWC_DIFF_, the difference in AnWC; TE, absolute typical error (in parenthesis: typical error expressed as a percentage of the grand mean); ES, Hedges’s *g*_*av*_ effect size (*Hg*_*av*_), 7 + Y_LIN_ and 7-Y_LIN_, the 7 × 4-min linear regression methods with the baseline metabolic rate as a Y-intercept either included (7 + Y) or excluded (7-Y); GE_LAST_, the gross efficiency model based on the last submaximal stage; CP_3´AO_, the critical power model based on the average 30-s end power during TT_AO_ as critical power. The same symbols used to illustrate individual data in Fig. 3 have also been used in this figure

Individual PO-MR regression data as based on the 7 + Y_LIN_ and 7-Y_LIN_ models together with GE calculated from the two linear regression equations for the submaximal stages and the TT, and measured GE, as well as the estimated AnWC for the 7 + Y_LIN_, 7-Y_LIN_, and GE_LAST_ models are shown in Fig. 3. Comparisons of the AnC/AnWC estimates from the 3-min TT_AO_ using the different models are presented in Table 2 and Fig. 4. As shown in Fig. 4A, the 7 + Y_LIN_ and 7-Y_LIN_ generated similar mean values of AnWC and the typical errors between the models were relatively low. The GE_LAST_ model generated significantly higher values (~ 12% higher) of AnWC than the 7 + Y_LIN_ and 7-Y_LIN_ models, due to the lower GE (see Table 2), and the typical errors were relatively low (2–3%) (Figs. 4B,C). The CP_3´AO_ model generated significantly lower values of AnWC (~ 30% lower, on average) than the 7 + Y_LIN_, 7-Y_LIN_, and GE_LAST_ models and the typical errors were high (23–26%) for all the respective comparisons (Figs. 4D–F).

**Fig. 5:**
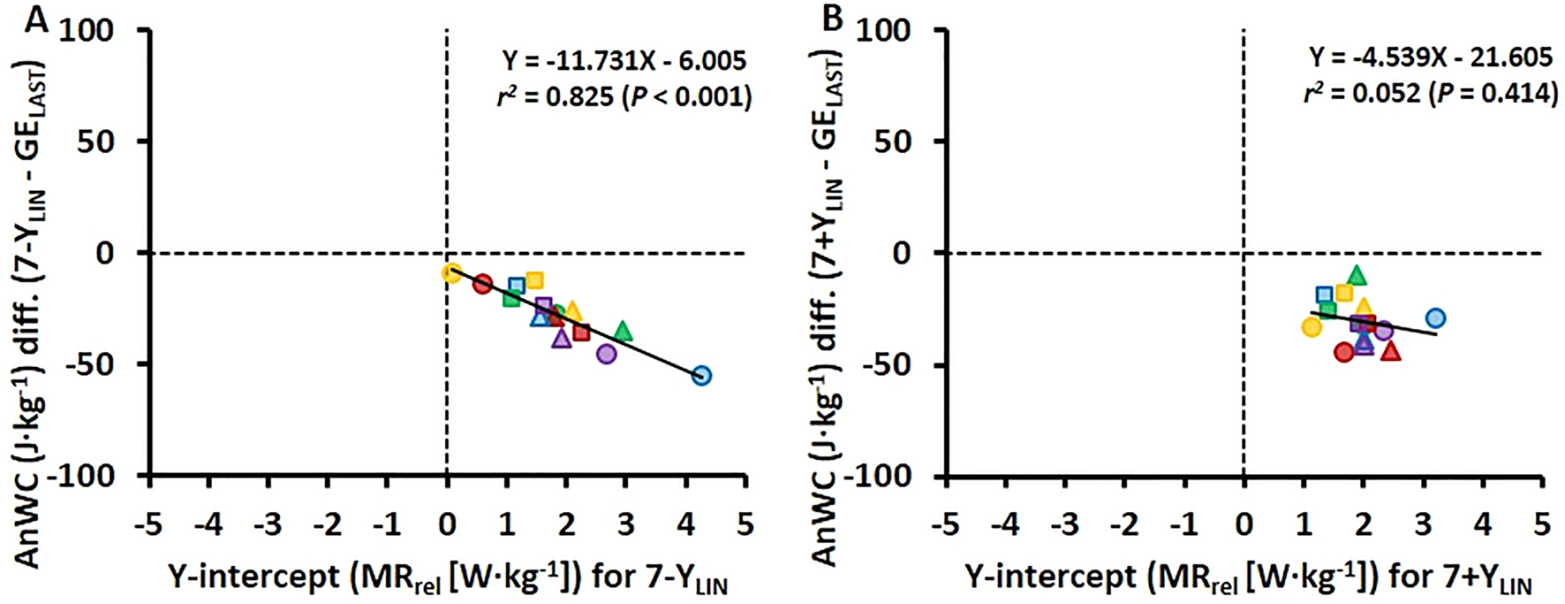
Scatter plots between the Y-intercept values for the 7 × 4-min linear regression models with the baseline metabolic rate (MR) as a Y-intercept either excluded (7-Y_LIN_) or included (7 + Y_LIN_) in the model (*x*-axis) and the anaerobic work capacity difference (AnWC diff.) versus the gross efficiency method based on the last submaximal stage (GE_LAST_) (*y*-axis)

The variation in Y-intercept values for the 7-Y_LIN_ model was highly related to the variation in the AnWC estimates between the 7-Y_LIN_ and GE_LAST_ models (*r*^2^ = 0.825; Fig. 5A), whereas the variation in Y-intercept values for the 7 + Y_LIN_ model was non-significantly related to the variation in the AnWC estimates between the 7 + Y_LIN_ and GE_LAST_ models (*r*^2^ = 0.052; Fig. 5B).

## Discussion

The main findings were as follows: 1) the GE_LAST_ model generated the highest AnWC/AnC estimate and the 7 + Y_LIN_ and 7-Y_LIN_ models generated similar average values of AnWC/AnC; 2) the typical errors in AnWC between the 7 + Y_LIN_, 7-Y_LIN_ and GE_LAST_ models were low; 3) the AnWC estimated with the CP_3´AO_ model was substantially lower and demonstrated considerably higher typical errors versus the 7 + Y_LIN_, 7-Y_LIN_, and GE_LAST_ models.

This is, to our knowledge, the first study that provides novel and detailed information on the within-participant (dis)agreement between four different models of estimating AnWC during cycle ergometry exercise. As hypothesized, the GE_LAST_ model generated significantly higher values of AnWC compared to the 7 + Y_LIN_ and 7-Y_LIN_ models (~ 12% higher), as well as to the CP_3´AO_ model (~ 63% higher). The higher AnWC estimates for GE_LAST_, when compared to 7 + Y_LIN_ and 7-Y_LIN_, could be explained by the positive Y-intercept in the 7 + Y_LIN_ and 7-Y_LIN_ regressions (Fig. 2A) and the increasing GE with higher power output (Fig. 2B), which is contrary to the GE_LAST_ model concept that assumes a constant GE. On average, this resulted in a 0.9 percentage point higher GE during the TT for the 7 + Y_LIN_ and 7-Y_LIN_ models versus the GE_LAST_ model. It is logical to suggest that the use of a Y-intercept value (i.e., resting baseline value of MR) in the linear PO-MR regression could only be justified if it is reasonably aligned with the submaximal stages of exercise. It appears that this is the case for the current study, because the slopes, delta efficiencies, and Y-intercept values were similar for the 7 + Y_LIN_ and 7-Y_LIN_ models. This finding is contrary to previous findings for other exercise modalities, such as diagonal-stride treadmill roller-skiing and treadmill running, where the inclusion of a Y-intercept lowered the slope of the regression line significantly and resulted in significantly lower estimates of AnC in highly trained participants (Andersson et al. 2021, 2020). Therefore, the exercise modality should be considered when deciding between the inclusion, or exclusion, of a baseline resting value as a Y-intercept in a linear regression model that is used for determining AnC (or AnWC).

In the current study, the typical errors for the AnWC estimates for the 7 + Y_LIN_, 7-Y_LIN_, and GE_LAST_ models were relatively low (2–4%). This could be partly explained by the relatively similar within-athlete regressions for the 7 + Y_LIN_ and 7-Y_LIN_ models (Figs. 3A, B). For instance, the Y-intercept values for the 7 + Y_LIN_ and 7-Y_LIN_ models were similar (see Figs. 2A and 3A, B). As observed previously (Andersson et al. 2020), the variation in Y-intercept values for the 7-Y_LIN_ model was highly related to the variation in the AnWC estimates between the 7-Y_LIN_ and GE_LAST_ models (see Fig. 5A). Since the Y-intercept values were all positive and showed a lower between-participants variation compared to previous observations for uphill diagonal-stride roller-skiing (and double poling) (Andersson et al., 2020), the typical errors for the GE_LAST_ versus 7 + Y_LIN_ and 7-Y_LIN_ models were also considerably lower compared to previous findings for other exercise modalities, such as roller-skiing and running (Andersson et al. 2021, 2020). Even though the typical errors for the GE_LAST_ versus 7 + Y_LIN_ and 7-Y_LIN_ models were relatively low (typical errors of 2–3%), it is probably still wise not to use different models interchangeably when testing athletes regularly.

Although some previous studies have compared and/or correlated different model estimates of AnWC/AnC derived from cycle-ergometry exercise trials (Dekerle et al. 2006; Green et al. 1994; Hill and Smith 1993; Muniz-Pumares et al. 2016; Noordhof et al. 2011), this is to our knowledge the first study that has compared AnWC estimates based on all the three main methods/concepts for estimating AnWC/AnC in endurance sports (Noordhof et al. 2013). In the current study, the AnWC associated with the CP_3´AO_ model was lower compared to the 7 + Y_LIN_, 7-Y_LIN,_ and GE_LAST_ models and showed substantial typical errors in AnWC versus the 7 + Y_LIN_, 7-Y_LIN,_ and GE_LAST_ models (see Figs. 4A–F). Further, the considerably lower AnWC estimate for the CP_3´AO_ model could be explained by the model assumption with CP representing a fixed (i.e., non-dynamic) aerobic contribution to PO and the similar aerobically attributable PO and CP during approximately the second half of TT_AO_ (see Figs. 2C,D). A similar result was found by Dekerle et al. (2006) for a 90-s all-out cycle test when comparing AnWC estimates based on a linear ${\dot{\text{v}}\text{o}}_{{2}}$-PO regression model with *W′* (i.e., “AnWC”) based on a conventional CP concept. Some previous studies have shown *W′*, based on any of the CP concepts, to be correlated with other method-specific estimates of AnWC/AnC, which suggests that *W′* is likely to represent an AnWC (Dekerle et al. 2006; Green et al. 1994; Hill and Smith 1993). However, Muniz-Pumares et al. (2016) compared AnC (expressed as an oxygen deficit) based on the MAOD method with the *W′* measure (i.e., “AnWC”) based on CP derived from the 30-s end power of a 3-min TT_AO_. The results showed that even if the AnC and *W′* measures were correlated (*r* = 0.654), there was still a poor agreement between the two measures, suggesting that one, or both, of the methods, was/were unable to accurately measure AnWC/AnC. Thus, both the study of Muniz-Pumares et al. (2016) and the results of the current study question both the validity and reliability of *W′* derived from a 3-min TT_AO_ as a measure of AnWC, that it was originally considered to represent (Hill 1993; Morton 2006).

Based on the average 30-s end power of TT_AO_, the CP was found to be 4.5 W kg^−1^, which for the 7 + Y_LIN_, 7-Y_LIN_, and GE_LAST_ models represented ~ 94% of the ${\dot{\text{v}}\text{o}}_{{{\text{2peak}}}}$ reached in TT_AO_. The current study was not designed to evaluate if the determined CP represented the maximal lactate (or metabolic) steady-state. However, based on previous research findings (Bartram et al. 2017; Iannetta et al. 2020; Karsten et al. 2014; Mattioni Maturana et al. 2016; Sperlich et al. 2011) and that the estimated exercise intensity of CP was 94% of ${\dot{\text{v}}\text{o}}_{{{\text{2peak}}}}$, it is likely that the CP was higher than the maximal lactate (or metabolic) steady state. This indicates that the 3-min TT_AO_ overestimated CP and, as a result, underestimated *W′*, i.e., the “AnWC” parameter, most likely also with questionable reliability (Bartram et al. 2017; Karsten et al. 2014). This could explain, at least in part, some of the substantial mean differences in AnWC between the CP_3´AO_ model versus the 7 + Y_LIN_, 7-Y_LIN_, and GE_LAST_ models that were observed in the current study. These results suggest that the *W’* estimate determined from the CP_3´AO_ model is likely to be less valid and reliable as an AnWC compared to the 7 + Y_LIN_, 7-Y_LIN_, and GE_LAST_ models.

It is important to bear in mind that *W’*, based on the CP_3´AO_, and AnWC, as based on any of the three other models (i.e., 7 + Y_LIN_, 7-Y_LIN_, and GE_LAST_), entails quite different methodological concepts. An important factor that is likely to explain the (dis)agreement between *W’* (i.e., “AnWC”) based on the CP_3´AO_ model and the other three models is rooted in the aerobic component of *W’*, which arises when aerobically attributable PO is in the intensity domain between CP and the maximal aerobically attributable PO (Vinetti et al. 2017; Vinetti et al. 2017). For example, if the average fractional utilization of ${\dot{\text{v}}\text{o}}_{{{\text{2peak}}}}$ (as a percentage of ${\dot{\text{v}}\text{o}}_{{{\text{2peak}}}}$) during a maximal effort is lower than the corresponding ${\dot{\text{v}}\text{o}}_{{2}}$ requirement at CP (as a percentage of ${\dot{\text{v}}\text{o}}_{{{\text{2peak}}}}$), *W’* would in theory attain a lower value than AnWC based on the GE_LAST_ model. This was the case in the current study and was partly caused by the overestimated CP. For similar average values of AnWC between the CP_3´AO_ model versus the GE_LAST_ model, the ${\dot{\text{v}}\text{o}}_{{2}}$ requirement at CP would have needed to be ~ 80% of ${\dot{\text{v}}\text{o}}_{{{\text{2peak}}}}$ because the average fractional utilization was ~ 80% of ${\dot{\text{v}}\text{o}}_{{{\text{2peak}}}}$ during TT_AO_.

As shown in Fig. 2C, the total PO and aerobic contribution to PO (calculated based on GE_LAST_) were approximately the same after 90 s of TT_AO_, which indicates that the AnWC/*W′* was depleted after ~ 90 s with a following plateau of PO during the later stages of the TT, similar to previous studies (Burnley et al. 2006; Vanhatalo et al. 2007). However, a potentially problematic aspect of the 3-min all-out TT concept when using 30-s average end power as a CP measure is related to the fundamental “non-pacing” characteristics, as it is suggested to be a consistent 3-min all-out effort, i.e., with no pacing involved (Burnley et al. 2006). This might be problematic and, thus, susceptible to an increased between-participants variation and decreased reliability, and/or validity, of 30-s end power as a measure of CP. In turn, this would decrease the reliability, and/or validity, of the CP_3´AO_ model for estimating *W’* (i.e., “AnWC”) (Bartram et al. 2017; Dotan 2022).

One factor that could have contributed to the overestimated CP during the 3-min TT_AO_ is the different bike settings that were used in the current study compared to the reference study by Burnley et al. (2006). In the current study, cadence and pedal torque were self-selected based on the use of normal shifters, whereas Burnley et al. (2006) used a linear factor set-up on the Lode ergometer (linear factor = power/cadence^2^), which means that torque increases linearly with cadence and that PO increases quadratically with cadence. The use of a linear factor could, potentially, trigger more of a true all-out maximal effort as PO increases quadratically with cadence and may result in a higher initial PO along with higher exhaustion in the second half of the TT and, thus, a more realistic 30-s end test CP. However, the self-selected cadence approach that was used in the current study resulted in a reasonable cadence for supramaximal exercise and it was only slightly higher than the cadence of 80–90 rev·min^−1^ that was used in the study by Burnley et al. (2006).

All models of estimating AnWC that are presented in the current study are based on some GE assumptions during exercise at a maximal effort. The GE_LAST_ model assumes a constant GE and the 7 + Y_LIN_ and 7-Y_LIN_ models assume either an increasing, a constant, or a decreasing GE with the given direction being set by the Y-intercept value in the linear regression (Andersson et al. 2020). Although the CP_3__´AO_ is not directly related to GE per se, the model is likely to assume a constant GE since CP is a constant entity, i.e., based on the assumptions that CP is the product of fixed values of aerobic MR and GE, or that aerobic MR and GE change inversely and proportionally so that CP remains constant. One apparent difference between the 7 + Y_LIN_, 7-Y_LIN_, and GE_LAST_ models is presented in Fig. 2B, where the 7 + Y_LIN_ and 7-Y_LIN_ models assume a slightly increasing GE with higher power outputs, whereas the GE_LAST_ model assumes a constant GE during the TT as based on the GE value from the last submaximal stage. Since some previous research findings show that GE declines during high-intensity exercise (de Koning et al. 2013; Noordhof et al. 2015; Sahlin et al. 2005), it is likely that both the 7 + Y_LIN_ and 7-Y_LIN_ models overestimated GE and consequently underestimated the AnWC/AnC. Therefore, the GE_LAST_ model might have generated a more valid measure of AnWC/AnC. This is also congruous with the results displayed in Fig. 2C and the all-out pacing concept with an early depletion of AnWC (Vanhatalo et al. 2007) and an approximately zero anaerobic power contribution (based on both GE_LAST_ and CP_3´AO_) during the second half of the TT. Although the decline in GE during supramaximal exercise is problematic to measure and includes several assumptions (de Koning et al. 2013), it is very likely that GE declines during high-intensity submaximal and/or supramaximal cycle exercise. This is probably related to a combination of factors, such as exercise hyperpnea (Dempsey et al. 1996), altered muscle recruitment patterns, and/or fatigue (Sahlin et al. 2005). All these factors are likely to explain most of the ${\dot{\text{v}}\text{o}}_{{2}}$ slow component during high- and supramaximal-intensity exercise (Sahlin et al. 2005). When considering all these factors, the most valid methodology for determining AnC/AnWC during cycle ergometry exercise for a group with relatively homogenous cardiovascular fitness (i.e., ${\dot{\text{v}}\text{o}}_{{{\text{2max}}}}$) is likely to be GE measured at a relatively high submaximal exercise intensity (~ 75% of ${\dot{\text{v}}\text{o}}_{{{\text{2max}}}}$). An alternative solution could be to use the 7 + Y_LIN_ or 7-Y_LIN_ model and determine a fixed GE value calculated from the PO-MR regression based on a MR that corresponds to ~ 75% of the maximal aerobic MR (i.e., ${\dot{\text{v}}\text{o}}_{{{\text{2max}}}}$ expressed as a MR). This GE value can then be used similarly to the GE_LAST_ model for determining AnC/AnWC.

This study provides practical information for exercise physiologists by demonstrating the (dis)agreement between different models of estimating AnWC/AnC. Exercise physiologists should be aware that the use of these models is not interchangeable. The optimal model is likely to be, at least to some extent, case-by-case dependent, however, this study suggests that particular caution should be used when interpreting *W’* from the CP_3’AO_ method as a measure of AnWC. As the test-to-test reliability of AnWC/AnC is of high practical importance to athletes/coaches, future research is needed to determine the test-to-test reliability of different models used for estimating AnWC/AnC.

In conclusion, the 7 + Y_LIN_ and 7-Y_LIN_ models generated 10% lower AnWC values than the GE_LAST_ model. This result was caused by the ~ 0.9 percentage points higher supramaximal GE for the 7 + Y_LIN_ and 7-Y_LIN_ (calculated based on the linear PO-MR regression). When expressed as an AnC, the same comparison generated 17% lower values for the 7 + Y_LIN_ and 7-Y_LIN_ models versus the GE_LAST_ model. Due to the similar slopes and Y-intercepts for the 7 + Y_LIN_ and 7-Y_LIN_ models, supramaximal GE and AnWC/AnC were the same for both models. The within-participants variation in AnWC estimates between the 7 + Y_LIN_, 7-Y_LIN_, and GE_LAST_ models was low, as indicated by the low typical errors that ranged between 7 and 11 J∙kg^−1^ (or 2–4% of the respective pair-wise grand mean). The CP_3´AO_ model generated the least valid and reliable estimate of AnWC, as revealed by the 30% lower AnWC values (on average) compared to the other models, and the substantial between-models typical errors (55–59 J∙kg^−1^, or 23–26% of the respective pair-wise grand mean).

## Data Availability

Data are available on request.

## Abbreviations

7 + Y_LIN_: The linear regression model between power output and metabolic rate used to estimate the required instantaneous metabolic rate during the time trial, including a baseline value of metabolic rate as a Y-intercept
7-Y_LIN_: The linear regression model between power output and metabolic rate used to estimate the required instantaneous metabolic rate during the time trial, excluding a baseline value of metabolic rate as a Y-intercept
AnC: Anaerobic capacity
AnWC: Anaerobic work capacity
MR_BL_: Baseline metabolic rate
CO_2_: Carbon dioxide
CP: Critical power
CP_3’AO_: The critical power method for determining anaerobic work capacity based on the 30-s average end power during the 3-min all-out time trial as critical power
ES_*Hg_av*_: Hedges’ *g* effect size
GE: Gross efficiency
GE_LAST_: Gross efficiency from the last submaximal stage used to estimate the required instantaneous metabolic rate during the time trial
GE_REG_: Gross efficiency calculated from the linear regression equation between power output and metabolic rate
MAOD: Maximal accumulated oxygen deficit
MR: Metabolic rate
MR_AE_: Aerobic metabolic rate
MR_AN_: Anaerobic metabolic rate
MR_TT_req_: The required metabolic rate during the time trial
O_2_: Oxygen
PO: Power output
PO_AN_cont_: Anaerobic contribution to power output
PO_TT_: Power output during the time trial
RER: Respiratory exchange ratio
TT: Time trial
TT_AO_: The 3-min all-out time trial
$${\dot{\text{v}}\text{o}}_{{2}}$$: Oxygen consumption
${\dot{\text{v}}\text{o}}_{{{\text{2max}}}}$: Maximal oxygen consumption
${\dot{\text{v}}\text{o}}_{{{\text{2peak}}}}$: Peak oxygen consumption
W′: Mechanical work above critical power
